# Proton TOCSY NMR relaxation rates quantitate protein side chain mobility in the Pin1 WW domain

**DOI:** 10.1007/s10858-022-00400-5

**Published:** 2022-07-21

**Authors:** Gaddafi I. Danmaliki, Peter M. Hwang

**Affiliations:** 1 Department of Biochemistry, University of Alberta, Edmonton, AB T6G 2H7 Canada; 2 Department of Medicine, University of Alberta, Edmonton, AB T6G 2R3 Canada

**Keywords:** Solution nuclear magnetic resonance, Protein side chain dynamics, ^1^H NMR relaxation rates, Dihedral angle, ^3^J couplings

## Abstract

Protein side chain dynamics play a vital role in many biological processes, but differentiating mobile from rigid side chains remains a technical challenge in structural biology. Solution NMR spectroscopy is ideally suited for this but suffers from limited signal-to-noise, signal overlap, and a need for fractional ^13^C or ^2^H labeling. Here we introduce a simple strategy measuring initial ^1^H relaxation rates during a ^1^H TOCSY sequence like DIPSI-2, which can be appended to the beginning of any multi-dimensional NMR sequence that begins on ^1^H. The TOCSY RF field compels all ^1^H atoms to behave similarly under the influence of strong coupling and rotating frame cross-relaxation, so that differences in relaxation rates are due primarily to side chain mobility. We apply the scheme to a thermostable mutant Pin1 WW domain and demonstrate that the observed ^1^H relaxation rates correlate well with two independent NMR measures of side-chain dynamics, cross-correlated ^13^C relaxation rates in ^13^C^β^H_2_ methylene groups and maximum observable ^3^J couplings sensitive to the χ_1_ side chain dihedral angle (^3^J_Hα,Hβ_, ^3^J_N,Hβ_, and ^3^J_CO,Hβ_). The most restricted side chains belong to Trp26 and Asn40, which are closely packed to constitute the folding center of the WW domain. None of the other conserved aromatic residues is as immobile as the first tryptophan side chain of the WW domain. The proposed ^1^H relaxation methodology should make it relatively easy to measure side chain dynamics on uniformly ^15^N- or ^13^C-labeled proteins, so long as chemical shift assignments are obtainable.

**Supplementary Information:**

The online version contains supplementary material available at 10.1007/s10858-022-00400-5.

## Introduction

**Fig. 1:**
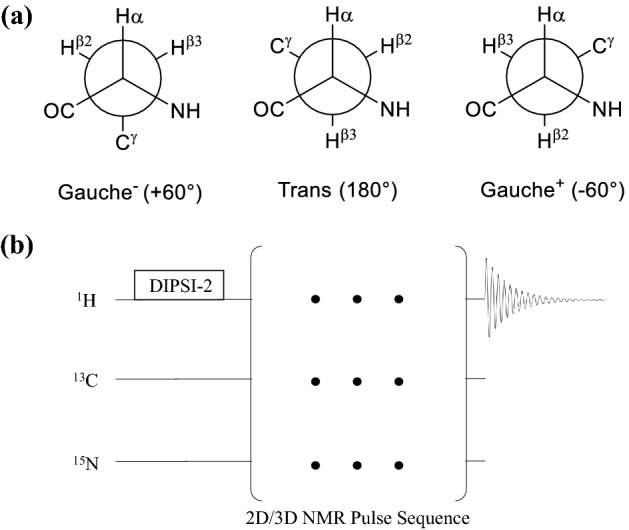
**a** The three major rotamers of the amino acid side chain χ_1_ dihedral angle. **b** Pulse scheme used to measure ^1^H relaxation rates, using any multi-dimensional sequence preceded by a.^1^H TOCSY sequence such as DIPSI-2

The power of nuclear magnetic resonance (NMR) spectroscopy lies in its ability to describe protein structure and how it changes as a function of time in solution at physiologic temperatures. Proteins are most mobile in their side chains, with a relatively rigid backbone except in the case of long loops and tails. In contrast, there is a high degree of variability between individual amino acid residues with respect to rotation along the Cα-Cβ bond defined by the χ_1_-dihedral angle. Steric considerations heavily favor three major rotamers (gauche + , gauche-, and trans), each pointing the side chain towards one of three corners of a tetrahedron (Fig. 1a). More than half of the side chains in a protein are mobile with respect to the χ_1_ dihedral angle (Mittermaier and Kay 2001; Li et al. 2015), but which ones are mobile or rigid may not be evident from a structural model derived from conventional X-ray, cryo-EM, or NMR data. A transition between χ_1_ rotamers can dramatically remodel the surface of a protein, potentially uncovering or obliterating a binding site, so it is important to know which side chains are mobile.

Traditionally, NMR has seen widespread application of ^15^N relaxation, because of its uniformity throughout the protein backbone (with the exception of proline residues) and ease of magnetically isolating the ^15^N nucleus. Discerning side-chain mobility by NMR is technically more challenging because of the diverse and interconnected ^1^H and ^13^C spin networks present in the 18 different amino acids with a structurally distinct χ_1_ dihedral angle (not glycine or alanine). Previous attempts to examine side chain relaxation have focused on ^2^H or ^13^C nuclei. ^2^H relaxation is appealing because the local quadrupolar relaxation mechanism dominates all others and can be used to map spectral densities that reflect the magnitude and timescale of dynamic fluctuations (Millet et al. 2002; Skrynnikov et al. 2002). The main drawbacks are the need for fractional deuteration and multiple magnetization transfer steps from ^1^H to ^13^C to ^2^H and back again. ^13^C relaxation studies typically use fractional ^2^H and ^13^C labeling to produce magnetically isolated ^13^C-^1^H or ^13^C-^1^H_3_ groups. Because of the development of labeling methods that produce near-100% ^13^C-^1^H_3_ labeled methyl groups in an otherwise ^12^C, ^2^H background, methyl group relaxation has become dominant in ^13^C relaxation studies (Tugarinov and Kay 2005). Of course, the drawback of this strategy is the lack of information about non-methyl-containing amino acids.

An alternative approach to studying side-chain dynamics is cross-correlated relaxation in ^13^C^1^H_2_-methylene groups, related to the ratio of signal intensities within the ^13^C^1^H_2_ multiplet (triplet) (Yang et al. 1998; Zheng and Yang 2004). The beauty of this approach is that it can be applied to a uniformly ^13^C,^15^ N-labeled sample without deuteration and easily incorporated into conventional 3-D pulse sequences that provide the needed spectral resolution to resolve most signals in the protein. Yang and Kay have demonstrated a reasonable correlation between relaxation parameters obtained using this method and the more rigorous ^2^H relaxation techniques (Yang et al. 1998). The drawback of this approach is the prolonged spin evolution time needed to separate out ^13^C^1^H_2_ multiplet components, substantially decreasing signal-to-noise relative to the original pulse sequences.

Herein we propose that the measurement of ^1^H relaxation rates, which, while complicated by remote ^1^H-^1^H interactions, provides the most facile and versatile means for probing side chain dynamics in uniformly ^15^ N- and/or ^13^C-labeled protein. ^1^H relaxation is complex because of multiple ^1^H-^1^H interactions, mediated via through-bond J couplings and through-space dipolar interactions. Both mechanisms are dependent on the chemical shift difference between interacting ^1^H spins. J coupling evolution can proceed via “weak” or “strong” coupling, depending on the magnitude of the J coupling relative to the chemical shift difference. As well, dipolar cross-relaxation for transverse magnetization, rotating-frame Overhauser enhancement (ROE), increases in contribution as the chemical shift difference between interacting sites decreases. Because of these effects, it is preferable to apply a radiofrequency (RF) field so that all ^1^H nuclei in the protein behave similarly with respect to these phenomena, thus evolving with strong coupling and maximum ROE contribution. Moreover, since the purpose of the RF field is to achieve uniformity throughout the protein, it is preferable to use a mixing scheme that compensates for off-resonance effects, like DIPSI-2, so that all ^1^H nuclei in the protein evolve similarly regardless of their position in the spectrum (Fig. 1b).

In the DIPSI-2 mixing scheme, magnetization starts along the z-axis and is rotated repeatedly clockwise or counter-clockwise around the x-axis by applied RF fields. Then the z-component of the magnetization relaxes according to.

1 $$\frac{{d\Delta I_{z,1} \left( t \right)}}{dt} = - \rho_{z,1} \Delta I_{z,1} \left( t \right) - \sigma_{1,2}^{NOE} \Delta I_{z,2} \left( t \right)$$

where ${\Delta I}_{z,1}\left(t\right)= {I}_{z,1}\left(t\right)-{I}_{z,1}^{0}$ , and ${I}_{z,1}^{0}$ is the equilibrium magnetization of spin-1 along the + z-axis. ρ_z,1_ is the longitudinal auto-relaxation rate for spin-1 and ${\sigma }_{\mathrm{1,2}}^{NOE}$ is the longitudinal cross-relaxation rate for spin-1 as it depends on spin-2. In the slow tumbling limit, where ${\omega }_{0}{\tau }_{c} \gg 1$,

2 $$\sigma_{1,2}^{NOE} = - \rho_{z,1} = \frac{{ - \hbar^{2} \mu_{0}^{2} \gamma_{H}^{4} \tau_{c} }}{{160\pi^{2} r_{H1,H2}^{6} }}$$

Thus, when spins 1 and 2 have the same direction and magnitude, the longitudinal auto-relaxation term $-{\rho }_{1}{\Delta I}_{z,1}\left(t\right)$ cancels out the cross-relaxation term, ${\sigma }_{\mathrm{1,2}}^{NOE}\Delta {I}_{z,2}\left(t\right)$, which is to say that longitudinal relaxation effects are very small when all the ^1^H nuclei (including H_2_O) start off along the + z-axis and evolve similarly under the influence of the DIPSI-2 mixing sequence.

For the transverse components of the magnetization,

3 $$\frac{{dI_{y,1} \left( t \right)}}{dt} = - \rho_{y,1} I_{y,1} \left( t \right) - \sigma_{1,2}^{ROE} I_{y,2} \left( t \right)$$

In contrast to the situation for the longitudinal component magnetization, for the transverse magnetization component, the auto- and cross-relaxation terms have the same (rather than opposite) signs. ${\sigma }_{\mathrm{1,2}}^{ROE}$ has a sign opposite to ${\sigma }_{\mathrm{1,2}}^{NOE}$ and double the magnitude (Bothner-By et al. 1984):

4 $$\sigma_{1,2}^{ROE} = \frac{{4\rho_{y,1} }}{5} = \frac{{\hbar^{2} \mu_{0}^{2} \gamma_{H}^{4} \tau_{c} }}{{80\pi^{2} r_{H1,H2}^{6} }}$$

Thus, the effect of cross-relaxation is to approximately double the observed transverse relaxation rate relative to auto-relaxation (rather than canceling out to zero as for longitudinal relaxation) (Bothner-By et al. 1984). During a DIPSI-2 mixing sequence, magnetization is rotated around the x-axis, wherein transverse auto- and cross-relaxation (ROE) dominate the relaxation of ^1^H spins.

Within a methylene CH_2_ group, the geminal dipolar interaction is at least 8 times stronger than any other ^1^H-^1^H interactions due to the r^−6^ distance dependence (1.8 Å distance between geminal protons versus > 2.55 Å for vicinal protons). Thus, due to cross-relaxation, geminal proton pairs within a single methylene group would be expected to have very similar relaxation rates. While “mixing” also occurs with respect to more remote proton pairs, the impact of more remote protons is minor compared to the local interactions within a single CH_2_ group.

As the spins evolve under the influence of the applied RF field, strong J-coupling also causes magnetization transfer between interacting spins (Bax 1989):

5 $$I_{z,1} \left( t \right) \to \frac{1}{2}\left( {I_{z,1} \left( {1 + \cos 2\pi Jt) + I_{z,2} (1 - \cos 2\pi Jt} \right)} \right) + \frac{1}{2}(2I_{y,1} I_{x,2} - 2I_{x,1} I_{y,2} )\sin 2\pi Jt$$

6 $$I_{z,2} \left( t \right) \to \frac{1}{2}\left( {I_{z,2} \left( {1 + \cos 2\pi Jt) + I_{z,1} (1 - \cos 2\pi Jt} \right)} \right) + \frac{1}{2}(2I_{y,2} I_{x,1} - 2I_{x,2} I_{y,1} )\sin 2\pi Jt$$

Similar equations can be written for magnetization components along the x- and y-axes. It will be noted that if ${I}_{z,1}$ and ${I}_{z,2}$ are both present at their equilibrium populations at time zero and both spins have the same relaxation rates, then there is no net transfer of magnetization. However, if the two spins have different relaxation rates, then net magnetization transfer will occur as the magnetization of the two spins begin to diverge in magnitude. As pointed out above, the geminal protons in a CH_2_ group will have nearly identical transverse relaxation rates due to cross-relaxation, so the majority of magnetization transfer due to strong coupling will be between vicinal protons. The maximum ^1^H-^1^H ^3^ J coupling observed in proteins is about 14 Hz, so in this case, according to Eqs. (5) and (6), magnetization would be fully interchanged between two strongly coupled spins by time 1 / (2 J) = 36 ms. Thus, we recommend measuring relaxation rates during time intervals that are much shorter than this, and ideally less than 18 ms, when the strong coupling-mediated transfer reaches its maximal rate. Moreover, we recommend only measuring initial relaxation rates (for instance, so that the most rapidly relaxing ^1^H signals do not decay to less than half of their starting signal) to further minimize magnetization transfer. Shorter relaxation delays < 18 ms can be employed as the size of the protein system increases. Thus, the confounding effect of strong coupling becomes less pronounced as larger systems are used, though we demonstrate that this methodology is effective in the smallest of protein domains. To demonstrate the validity of this approach, we measure ^1^H R_DIPSI-2_ relaxation rates in the human Pin1 WW domain. We also compare ^1^H relaxation rates of β-CH_2_ methylene groups with their corresponding 3-bond J couplings that are sensitive to the χ_1_-dihedral angle, ^3^J_Hα,Hβ_, ^3^J_N,Hβ_, and ^3^J_CO,Hβ_. The ^1^H relaxation rates and J couplings attain maximum values when the protein side chain is rigid, whereas rapid rotation about the χ_1_-dihedral angle markedly decreases both. Correlation between these two independent measures of side chain mobility indicates that ^1^H relaxation rates, like J couplings, can be a reliable measure of side chain mobility.

## Materials and methods

A mutant form of human Pin1 WW domain with N-terminal His-tag was overexpressed in *E. coli* bacteria and purified as previously described, with uniform ^15^N or ^15^N,^13^C isotope enrichment (Danmaliki et al. 2017). For NMR samples, protein concentration was 1 mM, with 10 mM imidazole, pH 6.7, 100 mM KCl.

Standard Varian VnmrJ Biopack NMR experiments were run at 30 °C using a Varian Inova 500 MHz spectrometer equipped with z-axis gradients and room temperature triple resonance probe.

For J coupling measurement experiments, the 3D out-and-back HNHB experiment was performed on an ^15^N-labeled sample (Archer et al. 1991; Bax et al. 1994). The intensity of cross-peaks was proportional to ${sin}^{2} (\pi Jt)$, where time t = 28 ms for evolution of the ^3^J_N,Hβ_ coupling, and this was used as a numerator in a ratio. A 2D HNHB control experiment in which ^15^N magnetization is not transferred to HB was used as the denominator. However, we decided not to use the data to calculate exact J couplings due to systematic error in comparing intensities in the 3D experiment with the 2D control. Instead, we elected to keep the ratio of the sine squared cross-peak to the diagonal peak control and present this normalized against the highest value found in the protein (which would correspond to the highest observed J coupling). Errors were estimated as the noise level divided by the cross-peak intensity.

We employed an approach like the HNHB experiment for the HN(CO)HB experiment (Grzesiek et al. 1992). Again, we maintained the ratio of the ${sin}^{2} (\pi Jt)$ cross-peak to the diagonal peak control. The time interval used for the evolution of the ^3^J_CO,Hβ_ coupling was 25 ms.

For the ^3^J_Hα,Hβ_ coupling, we used a different approach. We used the standard ^1^H-TOCSY-^13^C-HSQC 3D pulse sequence but modified the TOCSY mixing element from DIPSI-2 to DIPSI-2rc, which is modified to contain delays in which the magnetization is aligned along the z-axis, to cancel out ROE and NOE effects (Cavanagh and Rance 1992). Unlike the HNHB and HN(CO)HB, which are out-and-back experiments, the ^1^H-TOCSY-^13^C-HSQC experiment transfers magnetization to vicinal protons via strong coupling so that cross-peak intensity is dependent on $sin(2\pi Jt)$ rather than ${sin}^{2} (\pi Jt)$. It is not possible to run an equivalent 2D diagonal control spectrum as for HNHB and HN(CO)HB, so the data from this experiment is presented as the maximum observed ratio of Hβ2/Hβ3 or Hβ3/Hβ2 peak intensities.

For T_DIPSI-2_ experiments, a 5 kHz ^1^H DIPSI-2 pulse element was appended to the beginning of the 3D ^1^H-TOCSY-^15^ N-HSQC (22 ms mixing time, ^15^ N-labeled sample), 3D CBCACONH (22 ms mixing time, ^15^ N,^13^C-labeled sample in H_2_O buffer), 2D ^13^C-HSQC (11 ms mixing time, ^15^ N,^13^C-labeled sample in D_2_O buffer), or 3D ^1^H-TOCSY-^13^C-HSQC (11 ms mixing time, ^15^ N,^13^C-labeled sample in D_2_O buffer). We kept the ^1^H carrier frequency on water during the DIPSI-2 sequence, but upon further review, we think it would have been more appropriate to centre it at about 2.5 ppm to ensure better coverage of upfield ^1^H signals. A recycle delay of 2 s was used. Peak intensities were compared to the same experiment without the DIPSI-2 pulse element, and the ratio was used to calculate R_DIPSI-2_ rates. Like the J coupling data, these were also normalized to the fastest R_DIPSI-2_ rate found in the protein. Errors for the normalized values were estimated based on the expression

$$\sqrt {\left( {\frac{{\Delta_{1} }}{{I_{1} }}} \right)^{2} + \left( {\frac{{\Delta_{2} }}{{I_{2} }}} \right)^{2} }$$

where I_1_ and I_2_ are the intensities of the peaks used and $\Delta$_1_ and $\Delta$_2_ are the noise estimates of the associated spectra.

We recorded a 3D CBCA(CO)NH experiment to measure cross-correlation relaxation rates between ^1^H-^13^C dipoles of Cβ methylene groups using the indirect dimension to visualize the non-decoupled ^13^C triplet. The relaxation rates were calculated based on the deviation of the triplet intensities from 1:2:1 using the equation below (Yang et al. 1998; Zheng and Yang 2004; Yang 2011; Lesovoy et al. 2019).

7 $$\Gamma = - \frac{1}{4T} \ln \frac{{\left\{ {4 \times I_{out1} \times I_{out2} } \right\}}}{{\left\{ {I_{c} } \right\}^{2} }}$$

where ${I}_{\text{out1}}$, ${I}_{\text{out2}}$, and ${I}_{c}$ are the intensities of the outer lines 1 and 2 and the central line, respectively, and T is the constant time evolution period for the ^13^Cβ nucleus (22 ms), set to about 3/(4 J) for the one-bond C–C J coupling.

## Results and discussion

### Pin1 backbone dynamics

**Fig. 2:**
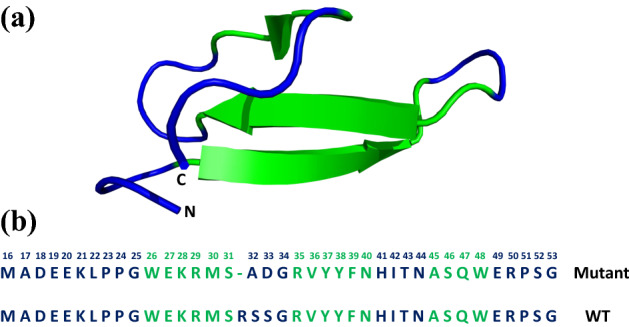
**a** Crystal structure of the human Pin1 protein (PDB id 2F21). **b** Sequence of the mutant Pin1 used in this study versus wild type. The antiparallel β-strands residues are highlighted in green

The mutant Pin1 WW domain protein used in this study contains 53 amino acid residues. The first 15 residues comprise a His-tag with a flexible Gly- and Ser-rich linker, which is not readily observable in the ^1^H-^15^N HSQC spectrum due to solvent-amide exchange and signal overlap. The native sequence begins at residue 16 in our construct. The structure comprises three antiparallel β-strands, with residues 22–53 visible in the X-ray crystal structure (Fig. 2) (Jäger et al. 2006). In agreement with the crystal structure, ^15^N T_1_ and T_2_ (from T_1ρ_) times indicate that the backbone of these residues is rigid throughout the WW domain, with significant mobility seen only N- and C-terminal to the structured core (residues 16–21 and 53) (Fig. S1). The similarity of backbone ^15^N T_1_ and T_2_ values indicate that this small protein (30-residue structured core) tumbles in the extreme narrowing regime with respect to ^15^N relaxation at 30 °C, wherein the spectral densities J(0) and J(ω_N_) are of similar magnitude, as opposed to the slow tumbling regime in which J(0) dominates.

### ^1^H RDIPSI_-2_ relaxation rates

We measured initial ^1^H relaxation rates during a DIPSI-2 time interval for the Pin1 WW domain, with normalized rates shown in Table 1.

**Table 1:** Chemical shifts and normalized values for maximum observed ^3^J-couplings and ^1^H R_DIPSI2_ relaxation rates for the mutant human Pin1 WW domain

Residue	Atom	Chemical shifts (ppm)	^3^J H_α_H_β_	^3^J HNH_β_	^3^J HN(CO)H_β_	^1^H R_DIPSI2_ from ^15^N TOCSY	^1^H R_DIPSI2_ from ^13^C TOCSY	^1^H R_DIPSI2_ from CBCACONH	Cross-correlated relaxation rates from CBCACONH(s^−1^)	Predominant Rotamer
M16	Hβ_2_/Hβ_3_	2.05/2.05	–	–	–	0.39	0.31	0.28	–	Mobile
A17	Hβ	1.42	^**a**^	–	–	0.28	0.16	0.18	–	
D18	Hβ_2_/Hβ_3_	2.64/2.74	0.42	0.35	0.34	0.31	0.29	0.34	−1.46	Mobile
E19	Hβ_2_/Hβ_3_	2.08/1.99	–	0.40	–	0.31	0.30	0.35	−2.17	Mobile
E20	Hβ_2_/Hβ_3_	2.03/1.83	–	–	–	0.42	^_^	0.31	−1.13	Mobile
K21	Hβ_2_/Hβ_3_	1.87/1.79	–	0.33	–	0.31	0.13	0.42	−1.66	Mobile
L22	Hβ_2_/Hβ_3_	1.45/1.85	^**a**^	^**b**^	–	0.74	0.71	–	–	Restricted
P23	Hβ_2_/Hβ_3_	2.63/2.02	0.45	–	–	–	0.79	–	–	Restricted
P24	Hβ_2_/Hβ_3_	1.91/2.36	0.39	–	–	–	0.60	0.53	−4.42	Restricted
G25	Hα_2_/Hα_3_	4.05/3.33	–	–	–	0.62	0.53	0.68	−7.20	
W26	Hβ_2_/Hβ_3_	3.27/2.99	1.00	1.00	–	0.89	0.82	0.88	− 10.64	Gauche+
E27	Hβ_2_/Hβ_3_	2.22/2.32	–	0.40	0.47	0.45	0.30	0.46	4.06	Mobile
K28	Hβ_2_/Hβ_3_	1.78/1.66	0.50	0.54	0.57	0.26	0.54	0.58	−2.53	Averaged between Trans and Gauche+
R29	Hβ_2_/Hβ_3_	0.09/1.32	0.70	0.79	0.27	0.69	0.70	0.78	−7.64	Gauche+
M30	Hβ_2_/Hβ_3_	1.98/1.89	0.38	0.41	0.50	0.16	0.28	0.37	0.30	Mobile
S31	Hβ_2_/Hβ_3_	4.53/4.23	–	–	–	–	0.22	–	–	Mobile
A32	Hβ	1.53	–	–	–	–	0.33	0.31	–	
D33	Hβ_2_/Hβ_3_	2.98/2.67	–	0.43	0.87	–	0.54	0.53	−5.85	Gauche-
G34	Hα_2_/Hα_3_	4.23/3.79	–	–	–	0.67	0.52	0.59	−5.34	
R35	Hβ_2_/Hβ_3_	1.96/2.05	–	–	–	0.32	–	0.63	–	Mobile
V36	Hβ	2.01	–	^**b**^	–	0.23	0.24	0.38	–	
	Hγ_1_/Hγ_2_	0.81/1.07					0.31/0.15			
Y37	Hβ_2_/Hβ_3_	2.46/2.79	0.39	0.89	0.95	–	1.00	1.00	−8.76	Gauche-
Y38	Hβ_2_/Hβ_3_	2.69/2.94	0.88	0.90	–	0.75	0.73	0.93	−6.40	Gauche+
F39	Hβ_2_/Hβ_3_	2.89/2.61	0.60	0.31	0.94	0.84	0.93	0.78	−7.98	Trans
N40	Hβ_2/Hβ3_	− 0.06/2.00	1.00	0.31	1.00	1.00	0.65	0.84	−6.97	Trans
H41	Hβ_2_/Hβ_3_	3.30/3.08	–	0.81	^**c**^	–	–	0.62	−2.15	Gauche-
I42	Hβ	2.02	^**a**^	^**b**^	–	–	0.46	0.42		
	Hγ_2_/Hδ_1_	0.79/0.76					0.30/0.12			
T43	Hβ	4.25	^**a**^	0.84	^**c**^	–	0.40	0.28	–	
	Hγ_21_	0.96					0.26			
N44	Hβ_2_/Hβ_3_	3.14/2.92	0.51	0.69	0.44	0.48	0.28	0.37	0.54	Averaged between Trans and Gauche+
A45	Hβ	1.26	^**a**^	–	–	0.22	0.26	0.26	–	
S46	Hβ_2_/Hβ_3_	3.84/3.79	–	0.46	0.40	0.43	0.67	0.47	0.54	Mobile
Q47	Hβ_2_/Hβ_3_	2.24/2.56	–	0.62	0.79	–	–	0.68	−12.02	Gauche-
W48	Hβ_2_/Hβ_3_	3.22/3.67	0.82	0.92	0.30	0.70	0.62	0.70	−9.03	Gauche+
E49	Hβ_2_/Hβ_3_	1.91/1.91	–	0.68	–	0.49	0.36	–	–	Mobile
R50	Hβ_2_/Hβ_3_	1.45/1.45	–	–	–	–	0.60	–	–	
P51	Hβ_2_/Hβ_3_	0.84/0.63	0.61	–	–	–	0.47	0.49	−2.87	Mobile
S52	Hβ_2_/Hβ_3_	3.81/3.75	0.36	0.30	0.45	0.49	0.35	0.26	−1.70	Mobile
G53	Hα_2_/Hα_3_	3.78/3.78	–	–	–	0.11	0.15		–	

Cβ cross-correlated relaxation rates have not been normalized. In the last column, the predominant rotamer is determined by comparing Hβ2 with Hβ3 intensities in the NMR experiments used to derive J couplings, but this is not always possible if the NMR signals are overlapped or degenerate. We label the residue “mobile” or “restricted” in such cases based on the available relaxation data. Generally speaking, “mobile” residues have normalized values < 0.55, while “restricted” residues have normalized values > 0.55. The value is left blank if the residues has no β CH2 group (A,G,I,V,T) or if there was insufficient data

^a^Single Hβ peak, but no second Hβ peak for quantitative ^3^J coupling estimation

^b^Peak present in HNHB spectrum, but the overlapped signal in 2D reference spectrum prevents quantitative ^3^J coupling measurement

^c^Peak present in HNCOHB spectrum but overlapped signal in 2D reference spectrum prevents quantitative ^3^J coupling measurement

The 3D ^1^H-TOCSY-^15^ N-HSQC experiment yielded ^1^H R_DIPSI2_ relaxation rates for most Hβ groups in the Pin1 WW domain. The experiment suffers from variable signal-to-noise depending on the size of the 3-bond ^1^H J couplings used to relay magnetization from Hβ to the backbone HN, smallest for the side chain χ_1_ gauche^−^ rotamer and for helical backbone ϕ dihedral angles. Fortunately, in the small β sheet WW domain, signal-to-noise was sufficient for most residues. However, for larger helical proteins, pulse sequences that transfer magnetization via ^13^C-^13^C couplings would be preferable. ^1^H R_DIPSI2_ relaxation rates vary substantially as one goes through the sequence of the Pin1 WW domain (Table 1, Fig. S2a). The most rapid ^1^H R_DIPSI2_ relaxation rate observed in the 3D ^1^H-TOCSY-^15^ N-HSQC belongs to Asn40 Hβ2/3, with a relaxation rate of 28.8 s^−1^, corresponding to a time constant of 34.7 ms. For comparison purposes, this rate was assigned a value of 1, and all other rates observed in the protein were normalized to this value. The Hβ protons of Asn40 have unique and divergent chemical shifts, consistent with a rigid side chain. Additional residues with rapid ^1^H R_DIPSI2_ relaxation rates include the following: Leu22 (0.74), Trp26 (0.89), Arg29 (0.69), Tyr38 (0.75), Phe39 (0.84), and Trp48 (0.70). These residues possess a rigid backbone structure, as indicated by the backbone ^15^N relaxation (Fig. S1). Hydrophobic aromatic residues (Phe, Trp, Tyr) possess some of the fastest relaxation rates, consistent with rigid side chains. Aromatic rings frequently make important contacts with both hydrophobic and polar residues. Changes in the side chain χ_1_ dihedral angle drastically re-position the bulky inflexible aromatic ring, making transitions less likely. Interestingly, all the rigid non-aromatic residues possess Hβ2 chemical shifts that are highly divergent from Hβ3 whereas mobile residues have more similar chemical shifts. It is likely that divergent methylene chemical shifts are a specific indicator for side chain rigidity, whereas similar methylene chemical shifts are not necessarily specific for mobility.

Glycine residues are unique in that they have a methylene CH_2_ group at the α position. According to backbone ^15^N relaxation (Fig. S1), Gly25 and Gly34 residues tumble like the rest of the WW domain, whereas Gly53 at the C-terminus is flexible. Gly25 and Gly34 have normalized ^1^H R_DIPSI2_ relaxation rates slower than the most rigid C^β^H_2_ groups, 0.62 and 0.67, respectively. This is reflective of there being fewer vicinal ^1^H-^1^H dipolar interactions as well as some increased mobility at the Gly positions, as gauged by ^15^N relaxation. The ^1^H R_DIPSI2_ relaxation rates at non-glycine Hα positions are generally slower than for Hβ methylene groups due to the absence of short-range dipolar ^1^H-^1^H interactions found in methylene groups (Table S1). Thus, relaxation rates at non-glycine Hα depend on vicinal and more distant ^1^H-^1^H interactions that depend on backbone and side chain dihedral angles, making them a less reliable indicator of backbone dynamics than ^15^N relaxation.

^1^H R_DIPSI2_ relaxation rates were also measured using the 3D ^1^H-TOCSY-^13^C-HSQC sequence, as shown in Table 1 and Fig. S2b. Tyr37 had the fastest relaxation rate of 32.6 s^−1^, and we normalized other relaxation rates in the protein against this value. Consistent with the 3D ^1^H-TOCSY-^15^ N-HSQC experiment, the same residues display rapid relaxation rates at the Hβ position: Leu22 (0.71) Trp26 (0.82), Arg29 (0.70), Tyr38 (0.73), Phe39 (0.93), Asn40 (0.65), and Trp48 (0.62). Additional sites in the protein are also accessible to this NMR experiment, suggesting some rigidity in the following residues as well: Pro23 (0.79), Pro24 (0.60), Tyr37 (1.00), and Arg50 (0.60). There are many more NMR signals obtainable via the 3D ^1^H-TOCSY-^13^C-HSQC experiment. For instance, proline residues are only accessible via the ^13^C experiments, as are additional sites further along the larger side chains. For the most part, the ^1^H relaxation rates along long side chains line up well with expectations, with slower relaxation rates observed as one moves further away from the backbone (Table S1).

We initially hoped that the 3D ^1^H-TOCSY-^13^C-HSQC experiment would yield better data than the 3D ^1^H-TOCSY-^15^N-HSQC experiment because of superior signal-to-noise. However, spectral distortions arising from the water peak and the ^1^H-^1^H diagonal make data obtained from the ^1^H-TOCSY-^13^C-HSQC experiment less reliable. It is also possible that the recycle delay of 2 s was not sufficient in the ^1^H-TOCSY-^13^C-HSQC, because protein ^1^H T_1_ relaxation is not driven by H_2_O magnetization along the + z-axis as it is for the ^1^H-TOCSY-^15^N-HSQC experiment. The strongest signal-to-noise, often by an order of magnitude, is seen in the diagonal peaks, but we found that data from these diagonal peaks are unreliable compared with that from cross-peaks or other data presented in this manuscript. Thus, for ^1^H relaxation rates derived from the 3D ^1^H-TOCSY-^13^C-HSQC experiment, we used the cross-peak with the strongest intensity if there was one above a minimum threshold. We averaged the value with other cross-peaks within 50% of that intensity, with the standard deviation of those values used for error bar estimation (see Fig. S2b).

Figure. S2c compares the relaxation rates derived from 3D ^15^N-TOCSY-HSQC against the ^13^C TOCSY-HSQC experiment. It is important to note that there were some discrepancies between the ^1^H R_DIPSI2_ relaxation rates measured using the 3D ^1^H-TOCSY-^13^C-HSQC versus the ^1^H-TOCSY-^15^ N-HSQC experiment, most notably for Lys28 Hβ (0.54 vs. 0.26), Asn40 Hβ (0.65 vs. 1.00), Asn44 (0.28 vs. 0.48), and Ser46 Hβ (0.67 vs 0.43). We suggest that for these discrepancies, the values obtained from the 3D ^1^H-TOCSY-^13^C-HSQC experiment are less reliable, as discussed above.

**Fig. 3:**
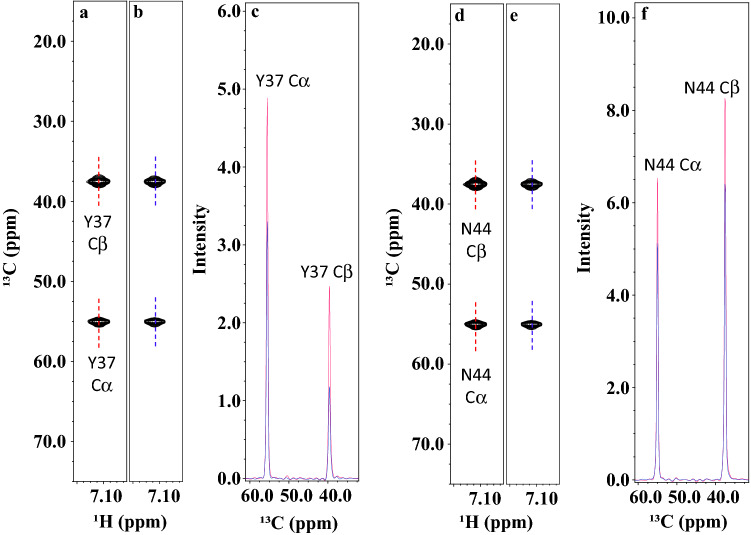
Effect of a DIPSI-2 relaxation interval on rigid Y37 and rotamer-averaging N44 residues. CBCA(CO)NH strip plots taken at 0 ms (**a**), (**d**), and the corresponding region at 22 ms (**b**), (**e**). Cross-sections of the peaks highlighted in the strip plots are shown in (**c**) and (**f**)

Table 1 also shows ^1^Hβ R_DIPSI2_ relaxation rates measured from the CBCA(CO)NH experiment. The most rapid ^1^Hβ relaxation in Pin1 belongs to Tyr37 (32.6 s^−1^), and we normalized other values found in the protein against it (Fig. S2c). Consistent with both ^1^H relaxation experiments, the same residues Trp26, Arg29, Tyr38, Phe39, Asn40, and Trp48 display rapid R_DIPSI2_ relaxation rates of 0.88, 0.78, 0.93, 0.78, 0.84, and 0.70, respectively. Importantly, the CBCA(CO)NH experiment was unaffected by the factors limiting the ^13^C TOCSY-HSQC experiment, making data obtained from this experiment more reliable. We also measured Hα R_DIPSI2_ relaxation rates (Table S1). We compare spectra from the CBCACONH experiment in Fig. 3 to demonstrate relaxation during the DIPSI-2 mixing scheme for a rigid residue, Y37, and a flexible residue, N44. After 22 ms, the Cα and Cβ intensities of Y37 decreased by ~ 33% and ~ 50%, respectively, whereas both in N44 decreased by ~ 22%.

### Cross-correlated relaxation rates of Pin1 residues

Table 1 also displays cross-correlated relaxation rates Γ derived from a CBCA(CO)NH experiment without ^1^H decoupling during ^13^C evolution in the indirect ^13^C dimension. Γ values range from +4.06 s^−1^ for flexible residues like Glu27 down to −12.02 s^−1^ for Gln47 (−7.20 for Gly25 Hα – note that only glycine residues have comparable cross-correlated relaxation at the α position in this experiment). The methylene groups of the rigid residues: Trp26, Arg29, Tyr37, Tyr38, Phe39, Asn40, Trp48 show cross-correlated relaxation rates of −10.64, −7.64, −8.76, −6.40, −7.98, −6.97, and −9.03, while the flexible Glu27, Met30, Asn44, and Ser46 exhibit positive rates of +4.06, 0.30, 0.54, and 0.54. It is interesting to note that in the original CBCA(CO)NH cross-correlated relaxation experiment described by Kay and co-workers performed on drk SH3 domain, there were no positive cross-correlated relaxation rates, indicating that the outer components of the ^13^C triplet have slower relaxation rates than the central line. This would be expected for the case where there is no internal side chain mobility or limited side chain mobility. However, the presence of positive cross-correlated relaxation rates confirms the presence of very fast side chain rotamer transitions between the major rotamers, faster than the overall tumbling of the WW domain, so that the magnetic fields of anti-parallel ^1^H spins in the central methylene ^13^C transition destructively interfere, instead of the constructive interference one would expect based on the tetrahedral 109° angle between ^13^C-^1^H dipoles. One would expect that these rapid transitions occur in all proteins and not just in the WW domain as we have observed, except that in larger proteins (which includes all folded domains), the J(0) spectral density contributed by overall tumbling as described by the order parameter S^2^ becomes more dominant, so that only negative cross-correlated relaxation rates are observed, even for a domain as small as an SH3 domain (though it is about double the size of the WW domain). Interestingly, Zheng and Yang also observe positive cross-correlated relaxation rates in the flexible lysine side chains of intestinal fatty acid binding protein (Zheng and Yang 2004). For comparison to ^1^H R_DIPSI2_ relaxation rates, we converted the values to a linear scale of 0 (for Glu27) to 1 (for Gln47) in Fig. S3.

### χ_1_ dihedral angle estimated using maximum observed ^3^ J couplings

Accurate measurements of 3-bond scalar coupling constants from ^3^J_Hα-Hβ_, ^3^J_N-Hβ_, and ^3^J_CO-Hβ_ experiments are critical for stereospecific chemical shift assignment of β-methylene protons and determination of the side chain χ_1_ rotameric state. J couplings also provide an alternative independent method to assess the validity of using relaxation measurements to gauge conformational dynamics.

**Fig. 4:**
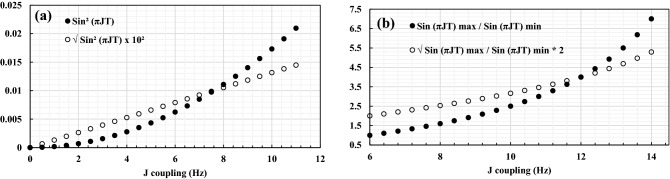
Correlation plot of simulated cross-peak intensity ratio against ^3^J couplings of HNHB/HN(CO)HB experiment (**a**) and HαHβ experiment (**b**). In both plots, taking the square root converts an exponentially shaped function to an approximately linear correlation with J-couplings

For measuring J couplings, we used quantitative methods that transfer magnetization from ^15^N or ^13^CO to Hβ_2_ and Hβ_3_ and then back. For instance, the three-bond J coupling ^3^J_N-Hβ_ is measured in the HNHB experiment by comparing the intensity ratio of the cross-peaks of the N-Hβ correlations in the 3D spectrum to the N-HN correlation in the 2D reference spectrum (similar amount of time spent on the ^15^N nucleus, but ^1^H-^15^N couplings refocused):

$$\frac{{I_{N - HB} }}{{I_{N} }} = sin^{2} \left( {\pi Jt} \right)$$

where *t* is the time spent evolving the ^15^N magnetization to Hβ_2/3_, with a second identical interval, *t*, evolving the magnetization from Hβ_2/3_ back to ^15^N. Since the couplings reported in the literature for N-Hβ or CO-Hβ are < 11 Hz (Archer et al. 1991; Grzesiek et al. 1992; Bax et al. 1994), we simulated the intensity ratio for 0 < J < 11 Hz. Figure 4a shows a parabolic correlation comparing the ${sin}^{2}\left(\pi Jt\right)$ term to J for t = 28 ms. By applying a square root, ${\surd sin}^{2}\left(\pi Jt\right)$, an approximately linear correlation can be achieved for this range of *Jt* values, because *sin πJt* ~ *πJt* for small values of *πJt*. Thus, we use ${\surd sin}^{2 }(\pi Jt)$ as proportional to the J coupling value and normalize the result against the highest value found in the protein. We chose not to estimate absolute values for the J couplings because this would introduce systematic error in comparing the 3D HNHB experiment with its 2D reference spectrum, and we are mainly interested in the normalized values for this study.

According to the Karplus equation, 3-bond J couplings achieve their maximum value when the interacting nuclei are oriented trans to each other and take on smaller values when they are at 60° (corresponding to the other major rotameric states). Since the accuracy of our J couplings measurements depends on signal intensity, we chose to focus on the maximum observable J coupling. The maximum observable values in the protein correspond to rigid side chains in which interacting nuclei are fixed in a trans orientation. With increasing mobility, the nuclei “wiggle” away from the trans position or jump to other rotameric states, decreasing the maximum observed J coupling, down to 33% in theory, assuming a minimum J coupling of around 0 Hz and complete averaging about the 3 major rotameric states.

For ^3^J_αβ_ couplings, we employed a ^1^H DIPSI-2rc TOCSY-^13^C-HSQC sequence to relay magnetization from Hβ to Hα and then to HN, in which delays are introduced into the DIPSI-2 pulse train while the magnetization is oriented along the z-axis in such a way that NOE and ROE cancel out (Cavanagh and Rance 1992). However, because this is not an out-and-back experiment, there is no 2D reference experiment. Thus, we used a different approach to estimating relative J-couplings by taking the larger of the cross-peak intensities (corresponding to Hβ_2_ or Hβ_3_) divided by the lesser intensity. The most rigid side chains give rise to the largest quotients, which tend towards 1 for rapid rotameric averaging. The ^3^J_αβ_ couplings previously reported in the literature range between < 4 Hz for gauche substituents and > 10 Hz for trans substituents, with an intermediate 6–8 Hz for disordered side-chains in equilibrium between two or three of the major rotamer conformations (Clore et al. 1991). Since the cross-peak intensity is dependent on $sin(2\pi Jt)$, we simulated the quotient $\mathrm{sin }\left(2\pi Jt\right)max/\mathrm{sin} \left(2\pi Jt\right) min$ versus J within the range 6 < J < 14 Hz, corresponding to rotameric averaging between 3 states, with the trans state having a theoretical J coupling of 14 Hz (max) and the gauche states, 2 Hz (min) (Fig. 4b). Taking the square root of the quotient $\mathrm{sin }\left(2\pi Jt\right)max/\mathrm{sin} \left(2\pi Jt\right) min$ converts an exponential-like function into a roughly linear correlation, with such a term being roughly proportional to ^3^J-couplings.

### The estimation of maximum measured ^3^J-coupling for Pin1 residues

We thus obtained measures proportional to ^3^J_N,Hβ_, ^3^J_CO,Hβ_, and ^3^J_α,β_ couplings and normalized them against the maximum values observed in the protein (Table 1 and Fig. S4). Residues with a single dominant χ_1_-rotamer are expected to display large couplings in two of the three J coupling measurement experiments, and the normalized couplings should be the same in theory (though for ^3^J_αβ_ compared to the others, this is only approximate). We excluded residues for which we could not obtain precise measurements due to signal overlap or poor signal-to-noise. Fig. S5 shows the normalized maximum ^3^J-coupling plotted against the human Pin1 sequence for residues with a complete data set. Consistent with the ^1^H relaxation experiments, Trp26 and Asn40 residues possessed the maximum observed ^3^J values (normalized to 1.0), confirming that the side chains of these residues are the most immobile in the entire protein.

**Fig. 5:**
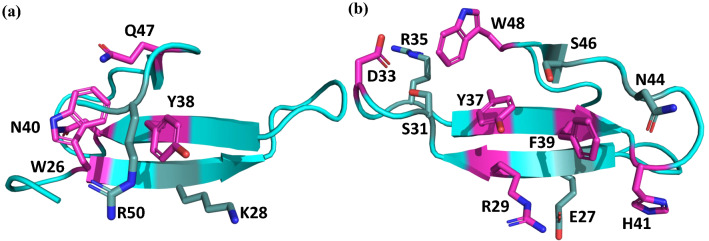
The crystal structure of the human Pin1 protein shows side chains for which NMR J-coupling experiments have defined a dominant χ_1_ dihedral angle. The side chains of Trp26, Tyr38, Asn40, and Gln47 are clustered on one side of the sheet as shown in (**a**), while side chains of Arg29, Asp33, Tyr37, Phe39, His41 and Trp48 constitute a surface on the other side as shown in (**b**). Flexible side chains interacting with the rigid residues are highlighted in light teal

Based on the J-couplings, we can differentiate residues into three groups: (1) rigid residues with a single dominant χ_1_ dihedral angle conformation (Trp26, Arg29, Asp33, Tyr37, Tyr38, Phe39, Asn40, His41, Gln47, and Trp48) as highlighted in Fig. 5; (2) residues averaging between two χ_1_ dihedral angle rotamers (Lys28 and Asn44); and (3) flexible residues averaging equally between all three χ_1_ dihedral angle rotamers (Met16, Asp18, Glu19, Glu20, Lys21, Glu27, Arg35, Ser46, Glu49, Ser52) (Table 1). Excluded from these groups are residues with no χ_1_ dihedral angle (Gly, Ala), residues without a β-methylene group (Ala, Thr, Ile, and Val), and any residues lacking sufficient data (Pro and others).

### Rigid Pin1 residues adopting a single dominant χ_1_ dihedral angle rotamer

**Fig. 6:**
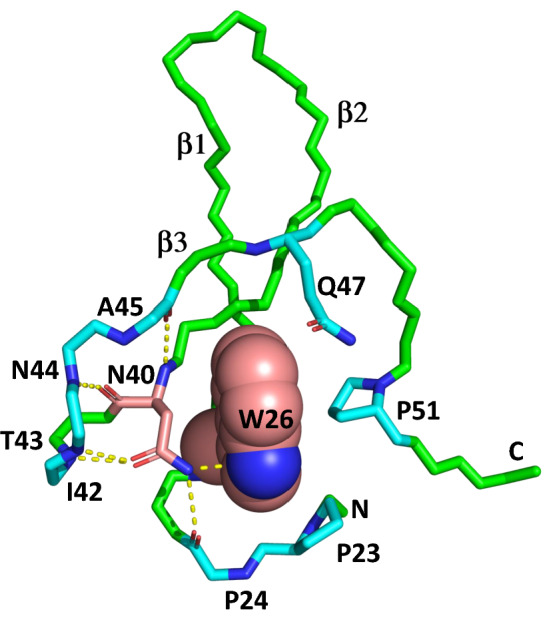
The folding core of the human Pin1 WW domain shows the buried residues: Trp26 (spheres) and Asn40 (salmon sticks). Backbone atoms of Pro24, Ile42, Thr43, Asn44, and Ala45 forming yellow hydrogen bonds with Asn40 are shown in cyan, as are the side chains of residues closely interacting with Trp26 (Pro23, Gln47, and Pro51)

According to ^3^ J coupling experiments, the most restricted side chains in the Pin1 WW domain belong to Trp26 and Asn40. The side chain of Asn40 is packed against the plane of the Trp26 indole ring, giving Asn40 the most unique and upfield-shifted Hβ chemical shifts in the entire protein (−0.60 and 2.00 ppm). The side chain amide HN of Asn40 hydrogen bonds with the π-cloud above the plane of the Trp26 indole ring nitrogen (Zhang et al. 2005), as shown in Fig. 6. The other HN forms a hydrogen bond with the backbone carbonyl of Pro24. The side chain amide oxygen of Asn40 additionally forms two strong hydrogen bonds with the backbone amide HN of Ile42 and Thr43. Thus, the side chain of Asn40 forms no fewer than four hydrogen bonds, three of which are with the backbone. The backbone of Asn40 hydrogen bonds with the backbone HN of Asn44 and Ala45, forming an unusual 5-residue short loop structure that connects the second and third β-strands. The Trp26-Asn40 dyad, sitting on the first two β-strands, constitutes the central folding core of the WW domain (see Fig. 6). The indole side chain of Trp26, which belongs to the first β-strand, forms packing interactions with Gln47 of the third β-strand, and Pro23 and Pro51 from the N- and C-terminal tails of the WW domain. Thus, all three β-strands and the N-terminal and C-terminal tails of the small WW domain appear to converge around the first tryptophan side chain for which the domain is named. Thus, the maximum χ_1_-sensitive ^3^J couplings observed in the protein highlight the Trp26-Asn40 dyad that defines the folded core of the Pin1 WW domain (Fig. 6).

In contrast to Trp26, Trp48 is relatively solvent-exposed and displays more mobility than Trp26, as indicated by all relaxation and J coupling measurements (Table 1), though both have a dominant gauche^+^ rotamer. The side chains of the aromatic residues, Tyr37, Tyr38, and Phe39, appear to be rigid by both J couplings and relaxation, and their aromatic rings have significant packing interactions with polar side chains as well as remote regions of the backbone: the aromatic ring of Tyr37 contacts Arg29, Ser31, and Ser46; Tyr38 contacts Lys28 and Arg50; and Phe39 contacts Arg29, His41, Asn44, and Ser46 (Fig. 5). The hydrophilic side chains are mobile, as assessed by ^1^H relaxation and/or J couplings, except for Arg29. Besides contacting two aromatic residues, Arg29 also forms a salt bridge with Glu27. Arg29 also has a unique Hβ2 chemical shift at 0.09 ppm due to its close approach to Tyr37.

It is noteworthy that the side chain of Asp33 is rigid, even though this residue is not part of the native Pin1 WW domain sequence. Asp33 is part of a deletion-substitution mutation found to stabilize the WW domain (Jäger et al. 2006). The side chain of Asp33 is in the sterically disfavored gauche^−^ conformation (Fig. S6), which allows its carboxyl group to hydrogen bond with the side chain of Ser31, with both side chains forming electrostatic interactions with the ring protons of Trp48.

**Fig. 7:**
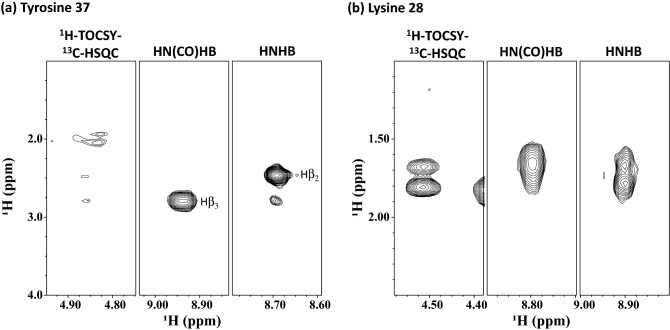
NMR strip plots from 3D ^1^H-TOCSY-^13^C-HSQC, HN(CO)Hβ, and HNHβ for a rigid Pin1 residue, Tyr37, adopting the gauche- χ_1_ rotamer (a), and flexible residue Lys28 sampling two χ_1_ rotamers (b). ^1^H-TOCSY-^13^C-HSQC and HNHβ strips are taken at the Hβ and NH frequencies, respectively, of residue i, while HN(CO)Hβ strips are taken at the NH frequency of residue i + 1. The intensity of each correlation relates to the size of each ^3^J coupling

Figure 7a shows strip plots from 3D ^1^H-TOCSY-^13^C-HSQC, HNCOHβ, and HNHβ for Tyr37. The residue predominantly adopts the gauche^−^ χ_1_-rotamer with downfield resonance assigned to Hβ_3_. For comparison, strip plots for a side chain with rotameric averaging, Lys28, are shown in Fig. 7b.

### Pin1 residues averaging between two χ_1_ dihedral angle rotamers

**Fig. 8:**
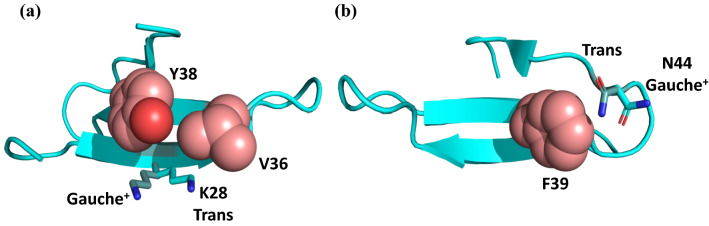
The two favored rotamers of Lys28 (**a**) and Asn44 (**b**). The trans/gauche^+^ rotamers are shown as sticks in cyan (crystal structure conformation) or light teal (alternative equally sampled rotamer by NMR). The residues contacting Lys28 (Val36 and Tyr38) and Asn44 (Phe39) are shown as salmon spheres

Based on J couplings, we could identify just two residues with rotameric mobility about two of the three major χ_1_-dihedral angle positions: Lys28 and Asn44. Without stereospecific assignments for Hβ2/3, both residues appear to have a slight preference for the sterically unfavorable gauche^−^ rotamer (see Fig. 7 for Lys28). However, with stereospecific assignments, it becomes clear that the residues are instead averaging between the trans and gauche^+^-rotamers (Fig. 8). We assigned stereospecific Hβ resonances using an isotope labelling scheme we developed previously, which selectively protonates the Hβ_2_ of Asp, Asn, Lys, and Met amino acid residues with deuteration at Hβ_3_ using fumarate as a carbon source for *E. coli* in D_2_O (Danmaliki et al. 2017). In the X-ray crystal structure, Lys28 is partially solvent-exposed on the first β-strand of Pin1 and interacts with Val36 and Tyr38 (Fig. 8). All 3 rotameric positions are accessible to Lys28, but the trans conformation allows it to interact more closely with Val36, while the gauche^+^ conformation brings it into closer contact with Tyr38. The relaxation rates of Lys28 clearly indicate that its side chain is mobile, whereas the J couplings are intermediate between those of rigid residues and those that can freely access all three major rotamers.

Asn44 is found in the loop connecting the second and the third β-strands of Pin1 (Fig. 2). The χ_1_ dihedral angle for Asn44 is gauche^+^ from the crystal structure. Like Lys28, all three χ_1_ dihedral angle rotamers are accessible to Asn44, but the gauche^+^ and trans rotamers allow its sidechain to form close contacts with the aromatic ring of Phe39 (Fig. 8). As with Lys28, relaxation data indicate that the side chain of Asn44 is mobile, but the maximum observed J couplings are intermediate between those of rigid and mobile residues. It is noteworthy that for both Lys28 and Asn44, the two preferred rotamers allow the hydrophilic side chains to make contacts with other side chains, whereas the disfavoured gauche^−^ rotamer is more solvent-exposed.

The examples of Lys28 and Asn44 demonstrate that for mobile residues, one should not attempt to define the χ_1_-dihedral angle without independently obtained stereospecific assignments for Hβ residues. It is impossible to distinguish between there being one preferred rotamer with substantial access to the other two major rotamers, versus the other two rotamers being preferred with the stereospecific Hβ assignments reversed. In such cases, it is possible to obtain the wrong stereospecific assignments with the wrong rotamer preferences.

### Methyl-containing residues

The alanine β-methyl group is rigidly attached to the backbone and should provide a gauge of backbone dynamics comparable to ^15^N. There are three alanine residues in our mutant Pin1 WW domain, Ala17, Ala32, and Ala45. Of these, Ala32 and Ala45 are part of the folded WW domain and have normalized relaxation rates of 0.33 and 0.26, respectively (Table 1). These values are perhaps higher than what might be expected for a rapidly spinning methyl group, given that other residues with larger side chains have comparable or even slower rates (for example, D18, K28, M30). This may be due to the fact that the initial relaxation rate of an alanine methyl group is dominated by its faster-relaxing components produced by constructive interference of the intra-methyl ^1^H dipoles, and this phenomenon is not present in β-methylene side chains. Similar relaxation rates are observed for the γ–methyl groups of Val36, Ile42, and Thr43, suggesting that the side chains of these residues may be immobile. In theory, it should have been possible to determine the dominant rotamer for these residues, but this was hampered by signal overlap. It is important to note that we were able obtain meaningful relaxation data for all 20 amino acid types via ^1^H relaxation: β-methylene positions for most amino acid residues, α-methylene positions for Gly, β-methyl positions for Ala, and γ-methyl positions for Val, Ile, and Thr.

### Correlations between maximum observed ^3^J couplings and protein side-chain relaxation rates

**Fig. 9:**
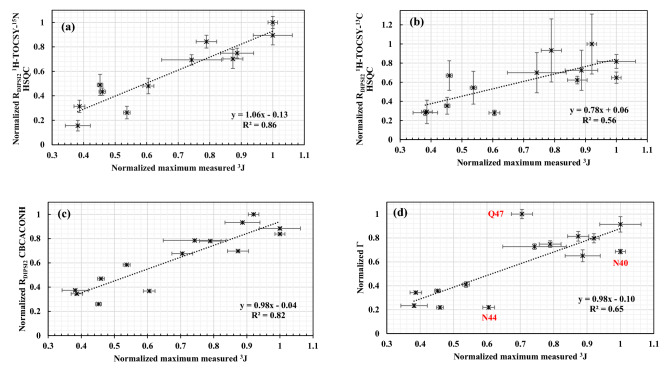
Correlation plot of the normalized maximum observed ^3^ J coupling and normalized R_DIPSI2_ derived from ^1^H-TOCSY-^15^N-HSQC (**a**), ^1^H-TOCSY-^13^C-HSQC (**b**), CBCACONH (**c**), and cross-correlated relaxation rates from CBCACONH (**d**). Errors bars are indicated on the vertical and horizontal axis

We have compared the measured ^1^H relaxation rates at Cβ derived from 3D ^15^N-TOCSY-HSQC with the maximum measured ^3^J-couplings in Fig. 9a. The figure shows a strong correlation, with a correlation coefficient of 0.86. Both independent mobility measures agree that the most rigid side chains in the entire Pin1 WW domain belong to the Trp26-Asn40 dyad at the folded center of the domain. The reason for the strong correlation is that rotation about the χ_1_-dihedral angle attenuates both maximum observed J couplings as well as transverse magnetization ^1^H relaxation rates. In theory, J coupling measurements are sensitive to a much larger range of motion timescales, everything from sub-nanosecond timescale motions up to tens of milliseconds (exchange broadening would become a confounding factor towards the slower end of this range). Thus, the fact that we observe a strong correlation between J couplings and ^1^H relaxation indicates that almost all the side chain rotameric averaging in Pin1 WW domain is happening on a very fast time scale. Further studies in other systems would be needed to demonstrate if this is generally applicable to proteins or not. We note that Arseniev and coworkers (Lesovoy et al. 2019) also observed a correlation between J couplings and relaxation for a small 61-residue water-soluble protein neurotoxin II.

Figures 9b–d, show positive correlations between the other relaxation experiments and maximum observed ^3^ J values. The correlations for the DIPSI-2-based relaxation data derived from the ^1^H-TOCSY-^13^C-HSQC (Fig. 9b) experiment are not as strong as we observed for the ^1^H-TOCSY-^15^ N-HSQC and CBCACONH (Fig. 9c) experiments. As we noted earlier, we suspect that one issue with the data obtained from the ^1^H-TOCSY-^13^C-HSQC was spectral distortion arising from the water signal. Another potential issue is that the 3D ^1^H-TOCSY-^13^C-HSQC and 2D ^13^C-HSQC experiments were performed on samples dissolved in D_2_O, so that T_1_ relaxation in the protein is driven not by bulk solvent but by the intrinsic proton T_1_ relaxation rates in the protein, which can be highly variable depending on local dynamics. Thus, the recycle delay of 2 s may not have been adequate for ^1^H T_1_ spin–lattice relaxation to uniformly return all protein ^1^H spins sufficiently close to their equilibrium positions prior to repeat scans, leading to aberrant relaxation during the DIPSI-2 element. Whatever the reason, relaxation data from the 3D ^1^H-TOCSY-^13^C-HSQC (Fig. 9b) and the 2D ^13^C-HSQC (not shown) were less reliable and yielded poorer correlations than data obtained from the 3D ^1^H-TOCSY-^15^N-HSQC and 3D CBCACONH experiments (Figs. 9a and 9c). This was disappointing to us, as the ^13^C-based spectra possessed far superior signal-to-noise. Further studies on protein ^1^H T_1_ relaxation times and optimal recycle delays are warranted. Another alternative would be to use H_2_O-dissolved samples and modified ^13^C-HSQC pulse sequences that preserve solvent magnetization along the + z-axis, although this would greatly worsen solvent signal suppression.

Finally, for the cross-correlated relaxation experiment based on CBCACONH, the correlation with maximum observed ^3^ J values was not as strong as some of the other methods we tested (Fig. 9d). One issue could be that constructive and destructive interference of the magnetic fields from the two ^13^C-^1^H dipoles results in a net magnetic field that is less sensitive to rotations about the χ_1_-dihedral angle and more sensitive to backbone dynamics.

Analyzing ^3^ J-coupling measurements to determine χ_1_ dihedral angle conformation has a rich history in NMR (Archer et al. 1991; Clore et al. 1991; Grzesiek et al. 1992; Bax et al. 1994). However, most analyses have relied on the simplifying assumption of a dominant trans, gauche-, or gauche+ conformation, even though most protein side chains are mobile, averaging between two or three χ_1_ dihedral angle rotamers (Hu and Bax 1997; Tuttle et al. 2013). Few studies have used J couplings to measure side chain dynamics, and even fewer have compared them to relaxation-based estimates of side chain dynamics (Chou et al. 2003; Hu et al. 2005; Smith et al. 2021). J coupling experiments suffer from reduced sensitivity due to their reliance on small ^3^J couplings, as well as an inability to obtain information on protein residues in which methylene protons have identical or very similar chemical shifts. ^1^H relaxation measurements thus provide high quality data on protein residues that would otherwise be inaccessible to J coupling-based analysis.

Precise characterization of side-chain dynamics has proven to be a challenging process. We show that protein side-chain motions can be characterized using a simple method of measuring ^1^H relaxation during a ^1^H TOCSY element, complementing the dynamic information derived independently from ^3^J-coupling measurements. Historically, neither ^1^H relaxation nor ^3^J couplings have been extensively used to probe side chain dynamics. We hope that with the implementation of ^1^H relaxation methodology, studies of side chain dynamics by solution NMR can become routine.

## Supplementary Information

Below is the link to the electronic supplementary material.

Supplementary file1 (DOCX 610 kb)

## References

[CR1] Archer SJ, Ikura M, Torchia DA, Bax A (1991). An alternative 3D NMR technique for correlating backbone 15N with side chain Hβ resonances in larger proteins. J Magn Reson.

[CR2] Bax A (1989). Homonuclear Hartmann-Hahn experiments. Methods Enzymol.

[CR3] Bax A, Vuister GW, Grzesiek S (1994). Measurement of homo- and heteronuclear J couplings from quantitative J correlation. Methods Enzymol.

[CR4] Bothner-By AA, Stephens RL, Lee JM (1984). Structure determination of a tetrasaccharide: transient nuclear Overhauser effects in the rotating frame. J Am Chem Soc.

[CR5] Cavanagh J, Rance M (1992). Suppression of cross-relaxation effects in TOCSY spectra via a modified DIPSI-2 mixing sequence. J Magn Reson.

[CR6] Chou JJ, Case DA, Bax A (2003). Insights into the mobility of methyl-bearing side chains in proteins from 3JCC and 3JCN couplings. J Am Chem Soc.

[CR7] Clore GM, Bax A, Gronenborn AM (1991). Stereospecific assignment of b-methylene protons in larger proteins using 3D 15N-separated Hartmann-Hahn and 13C-separated rotating frame Overhauser spectroscopy. J Biomol NMR.

[CR8] Danmaliki GI, Liu PB, Hwang PM (2017). Stereoselective deuteration in aspartate, asparagine, lysine, and methionine amino acid residues using fumarate as a carbon source for *E. coli* in D2O. Biochemistry.

[CR9] Grzesiek S, Ikura M, Marius Clore G (1992). A 3D triple-resonance NMR technique for qualitative measurement of carbonyl-Hβ J couplings in isotopically enriched proteins. J Magn Reson.

[CR10] Hu JS, Bax A (1997). Determination of φ and χ 1 angles in proteins from 13C–13C three- bond J couplings measured by three-dimensional heteronuclear NMR. How planar is the peptide bond?. J Am Chem Soc.

[CR11] Hu H, Hermans J, Lee AL (2005). Relating side-chain mobility in proteins to rotameric transitions: Insights from molecular dynamics simulations and NMR. J Biomol NMR.

[CR12] Jäger M, Zhang Y, Bieschke J (2006). Structure-function-folding relationship in a WW domain. Proc Natl Acad Sci U S A.

[CR13] Lesovoy DM, Dubinnyi MA, Nolde SB (2019). Accurate measurement of dipole/dipole transverse cross-correlated relaxation Γ2 in methylenes and primary amines of uniformly 13C / 15N -labeled proteins. J Biomol NMR.

[CR14] Li F, Grishaev A, Ying J, Bax A (2015). Side chain conformational distributions of a small protein derived from model-free analysis of a large set of residual dipolar couplings. J Am Chem Soc.

[CR15] Millet O, Muhandiram DR, Skrynnikov NR, Kay LE (2002). Deuterium spin probes of side-chain dynamics in proteins. 1. Measurement of five relaxation rates per deuteron in 13C-labeled and fractionally 2H-enriched proteins in solution. J Am Chem Soc.

[CR16] Mittermaier A, Kay LE (2001). Χ1 Torsion angle dynamics in proteins, from dipolar couplings. J Am Chem Soc.

[CR17] Skrynnikov NR, Millet O, Kay LE (2002). Deuterium spin probes of side-chain dynamics in proteins. 2. Spectral density mapping and identification of nanosecond time-scale side-chain motions. J Am Chem Soc.

[CR18] Smith LJ, van Gunsteren WF, Hansen N (2021). On the use of side-chain NMR relaxation data to derive structural and dynamical information on proteins: a case study using hen lysozyme. ChemBioChem.

[CR19] Tugarinov V, Kay LE (2005). Methyl groups as probes of structure and dynamics in NMR studies of high-molecular-weight proteins. Chem Bio Chem.

[CR20] Tuttle LM, Dyson HJ, Wright PE (2013). Side-chain conformational heterogeneity of intermediates in the escherichia coli dihydrofolate reductase catalytic cycle. Biochemistry.

[CR21] Yang D (2011). Probing protein side chain dynamics via 13C NMR relaxation. Protein Pept Lett.

[CR22] Yang D, Mittermaier A, Mok YK, Kay LE (1998). A study of protein side-chain dynamics from new 2H auto-correlation and 13C cross-correlation NMR experiments: application to the N-terminal SH3 domain from drk. J Mol Biol.

[CR23] Zhang RB, Somers KRF, Kryachko ES (2005). Hydrogen bonding to π-systems of indole and 1-methylindole: Is there any OH⋯Phenyl bond?. J Phys Chem A.

[CR24] Zheng Y, Yang D (2004). Measurement of dipolar cross-correlation in methylene groups in uniformly 13C-, 15N-labeled proteins. J Biomol NMR.

