# Supplementary material for: Proton TOCSY NMR relaxation rates quantitate protein side chain mobility in the Pin1 WW domain
Source: J Biomol NMR. 2022 Jul 21;76(4):121–35. doi: 10.1007/s10858-022-00400-5 (PMC9427894; doi:10.1007/s10858-022-00400-5)
Supplement: Supplementary file 1 — Supplementary file1 (DOCX 610 kb) [file 10858_2022_400_MOESM1_ESM.docx]

**Supplementary Information**

Journal of Biomolecular NMR

Proton TOCSY NMR relaxation rates quantitate protein side chain mobility in the Pin1 WW domain

Gaddafi I. Danmaliki^1^, Peter M. Hwang^1,2*^

^1^Department of Biochemistry, University of Alberta, Edmonton, Alberta, Canada T6G 2H7

^2^Department of Medicine, University of Alberta, Edmonton, Alberta, Canada T6G 2R3

* Correspondence to: Peter M. Hwang

780-492-4158, 780-492-7277 (Fax)

[phwang1@ualberta.ca](about:blank)

**(a)**

**(b)**

**Fig. S1** Backbone dynamics of Pin1 measured by ^15^N T_1_ (a) and ^15^N T_2_ (b) relaxation. Errors are indicated as vertical bars. T2 relaxation is calculated from T1ρ

**Table S1.** Chemical shifts and normalized values for maximum observed ^3^J-couplings and ^1^H R_DIPSI2_ relaxation rates for the mutant human Pin1 WW domain. Cβ cross-correlated relaxation rates have not been normalized. In the last column, the predominant rotamer is determined by comparing Hβ2 with Hβ3 intensities in the NMR experiments used to derive J couplings, but this is not always possible if the NMR signals are overlapped or degenerate. In such cases, we label the residue “mobile” or “restricted” based on the available relaxation data. Generally speaking, “mobile” residues have normalized values <0.55, while “restricted” residues have normalized values >0.55. The value is left blank if the residues has no β CH2 group (A,G,I,V,T) or if there was insufficient data.

| **Residue** | **Atom** | **Chemical shifts (ppm)** | **^3^J H_α_H_β_** | **^3^J HNH_β_** | **^3^J HN(CO)H_β_** | **^1^H R_DIPSI2_ from ^15^N TOCSY** | **^1^H R_DIPSI2_ from ^13^C TOCSY** | **^1^H R_DIPSI2_ from CBCACONH** | **Γ_CβCα(CO)NH_ (s^-1^)** | **Predominant Rotamer** |
| --- | --- | --- | --- | --- | --- | --- | --- | --- | --- | --- |
|  |  |  |  |  |  |  |  |  |  |  |
| M16 | Hα | 4.51 |  |  |  | 0.11 | 0.21* | 0.27 |  |  |
|  | Hβ_2_ | 2.05 | **^_^** | **^_^** | **^_^** | 0.39 | 0.30 | 0.28 | **^_^** | Mobile |
|  | Hβ_3_ | 2.05 |  |  |  |  | 0.30 |  |  |  |
|  | Hγ_2_ | 2.48 |  |  |  |  | 0.13* |  |  |  |
|  | Hγ_3_ | 2.58 |  |  |  |  |  |  |  |  |
|  | Hε_1_ | 2.10 |  |  |  |  | -0.05* |  |  |  |
|  |  |  |  |  |  |  |  |  |  |  |
| A17 | Hα | 4.35 |  |  |  | 0.02 | 0.08 | 0.22 |  |  |
|  | Hβ | 1.42 | **^a^** | **^_^** | **^_^** | 0.28 | 0.16 | 0.18 | **^_^** |  |
|  |  |  |  |  |  |  |  |  |  |  |
| D18 | Hα | 4.6 |  |  |  | 0.10 | 0.16 | 0.29 |  |  |
|  | Hβ_2_ | 2.64 | 0.42 | 0.35 | 0.34 | 0.31 | 0.29 | 0.34 | -1.46 | Mobile |
|  | Hβ_3_ | 2.74 |  |  |  |  |  |  |  |  |
|  |  |  |  |  |  |  |  |  |  |  |
| E19 | Hα | 4.28 |  |  |  | 0.02 | 0.11 | 0.34 |  |  |
|  | Hβ_2_ | 2.10 | **^_^** | 0.4 | **^_^** | 0.31 | 0.30 | 0.35 | -2.17 | Mobile |
|  | Hβ_3_ | 1.99 |  |  |  |  |  |  |  |  |
|  | Hγ_2_ | 2.49 |  |  |  |  | 0.15 |  |  |  |
|  | Hγ_3_ | 2.49 |  |  |  |  | 0.15 |  |  |  |
|  |  |  |  |  |  |  |  |  |  |  |
| E20 | Hα | 4.29 |  |  |  | 0.13 | 0.15* | **^_^** |  |  |
|  | Hβ_2_ | 2.03 | **^_^** | **^_^** | **^_^** | 0.42 | **^_^** | 0.31 | -1.13 | Mobile |
|  | Hβ_3_ | 1.83 |  |  |  |  |  |  |  |  |
|  | Hγ_2_ | 2.31 |  |  |  |  | 0.23* |  |  |  |
|  | Hγ_3_ | 2.31 |  |  |  |  |  |  |  |  |
|  |  |  |  |  |  |  |  |  |  |  |
| K21 | Hα | 4.33 |  |  |  | 0.15 | 0.19 | **^_^** |  |  |
|  | Hβ_2_ | 1.87 | **^_^** | 0.33 | **^_^** | 0.31 | 0.13 | 0.42 | -1.66 | Mobile |
|  | Hβ_3_ | 1.79 |  |  |  |  |  |  |  |  |
|  | Hγ_2_ | 1.48 |  |  |  |  | 0.29 |  |  |  |
|  | Hγ_3_ | 1.48 |  |  |  |  |  |  |  |  |
|  | Hδ_2_ | 1.73 |  |  |  |  | 0.15 |  |  |  |
|  | Hδ_3_ | 1.73 |  |  |  |  |  |  |  |  |
|  | Hε_2_ | 3.04 |  |  |  |  | -0.01 |  |  |  |
|  | Hε_3_ | 3.00 |  |  |  |  |  |  |  |  |
|  |  |  |  |  |  |  |  |  |  |  |
| L22 | Hα | 4.33 |  |  |  | 0.23 | 0.32 | **^_^** |  |  |
|  | Hβ_2_ | 1.45 | **^a^** | **^b^** | **^_^** | 0.74 | 0.71 | **^_^** | **^_^** | Restricted |
|  | Hβ_3_ | 1.85 |  |  |  |  |  |  |  |  |
|  | Hγ | 1.87 |  |  |  |  | 0.42 |  |  |  |
|  | Hδ_11_ | 1.11 |  |  |  |  | 0.35 |  |  |  |
|  | Hδ_21_ | 0.83 |  |  |  |  | 0.37 |  |  |  |
|  |  |  |  |  |  |  |  |  |  |  |
|  |  |  |  |  |  |  |  |  |  |  |
|  |  |  |  |  |  |  |  |  |  |  |
| P23 | Hα | 4.87 |  |  |  |  | 0.47 | **^_^** |  |  |
|  | Hβ_2_ | 2.63 | 0.45 | **^_^** | **^_^** | **^_^** | 0.79 | **^_^** | **^_^** | Restricted |
|  | Hβ_3_ | 2.02 |  |  |  |  |  |  |  |  |
|  | Hγ_2_ | 1.81 |  |  |  |  | 0.51 |  |  |  |
|  | Hγ_3_ | 1.64 |  |  |  |  |  |  |  |  |
|  | Hδ_2_ | 3.01 |  |  |  |  | 0.69 |  |  |  |
|  | Hδ_3_ | 3.71 |  |  |  |  |  |  |  |  |
|  |  |  |  |  |  |  |  |  |  |  |
| P24 | Hα | 4.40 |  |  |  |  | 0.12 | 0.38 |  |  |
|  | Hβ_2_ | 1.91 | 0.39 | **^_^** | **^_^** | **^_^** | 0.60 | 0.53 | -4.42 |  |
|  | Hβ_3_ | 2.36 |  |  |  |  |  |  |  |  |
|  | Hγ_2_ | 2.18 |  |  |  |  | 0.43 |  |  |  |
|  | Hγ_3_ | 2.07 |  |  |  |  |  |  |  |  |
|  | Hδ_2_ | 3.94 |  |  |  |  | 0.64 |  |  |  |
|  | Hδ_3_ | 3.67 |  |  |  |  |  |  |  |  |
|  |  |  |  |  |  |  |  |  |  |  |
| G25 | Hα_2_ | 4.05 | **^_^** | **^_^** | **^_^** | 0.62 | 0.53 | 0.68 | -7.20 |  |
|  | Hα_3_ | 3.33 |  |  |  |  |  |  |  |  |
|  |  |  |  |  |  |  |  |  |  |  |
| W26 | Hα | 5.29 |  |  |  | 0.46 | 0.30 | 0.58 |  |  |
|  | Hβ_2_ | 3.27 | 1.00 | 1.00 | **^_^** | 0.89 | 0.82 | 0.88 | -10.64 | Gauche+ |
|  | Hβ_3_ | 2.99 |  |  |  |  |  |  |  |  |
|  |  |  |  |  |  |  |  |  |  |  |
| E27 | Hα | 4.88 |  |  |  | 0.46 | 0.36* | 0.48 |  |  |
|  | Hβ_2_ | 2.22 | **^_^** | 0.40 | 0.47 | 0.45 | 0.30 | 0.46 | 4.06 | Mobile |
|  | Hβ_3_ | 2.32 |  |  |  |  |  |  |  |  |
|  | Hγ_2_ | 2.57 |  |  |  |  | 0.26* |  |  |  |
|  | Hγ_3_ | 2.24 |  |  |  |  |  |  |  |  |
|  |  |  |  |  |  |  |  |  |  |  |
| K28 | Hα | 4.48 |  |  |  | 0.22 | 0.30 | 0.50 |  |  |
|  | Hβ_2_ | 1.78 | 0.50 | 0.54 | 0.57 | 0.26 | 0.54 | 0.58 | -2.53 | Averaged between Trans and Gauche+ |
|  | Hβ_3_ | 1.66 |  |  |  |  |  |  |  |  |
|  | Hγ_2_ | 1.13 |  |  |  |  | 0.49 |  |  |  |
|  | Hγ_3_ | 1.13 |  |  |  |  |  |  |  |  |
|  | Hδ_2_ | 1.73 |  |  |  |  | 0.25 |  |  |  |
|  | Hδ_3_ | 1.73 |  |  |  |  |  |  |  |  |
|  | Hε_2_ | 2.97 |  |  |  |  | 0.17 |  |  |  |
|  | Hε_3_ | 2.97 |  |  |  |  |  |  |  |  |
|  |  |  |  |  |  |  |  |  |  |  |
| R29 | Hα | 4.45 |  |  |  | 0.53 | 0.31* | 0.48 |  |  |
|  | Hβ_2_ | 0.09 | 0.70 | 0.79 | 0.27 | 0.69 | 0.70 | 0.78 | -7.64 | Gauche+ |
|  | Hβ_3_ | 1.32 |  |  |  |  |  |  |  |  |
|  | Hγ_2_ | 1.25 |  |  |  |  |  |  |  |  |
|  | Hγ_3_ | 1.41 |  |  |  |  | 0.60 |  |  |  |
|  | Hδ_2_ | 2.89 |  |  |  |  | 0.33 |  |  |  |
|  | Hδ_3_ | 2.61 |  |  |  |  |  |  |  |  |
|  |  |  |  |  |  |  |  |  |  |  |
| M30 | Hα | 5.31 |  |  |  | 0.39 | 0.28* | 0.41 |  |  |
|  | Hβ_2_ | 1.98 | 0.38 | 0.41 | 0.50 | 0.16 | 0.28 | 0.37 | 0.30 | Mobile |
|  | Hβ_3_ | 1.89 |  |  |  |  |  |  |  |  |
|  | Hγ_2_ | 2.61 |  |  |  |  | 0.16 |  |  |  |
|  | Hγ_3_ | 2.59 |  |  |  |  |  |  |  |  |
|  | Hε_1_ | 2.02 |  |  |  |  | -0.02* |  |  |  |
|  |  |  |  |  |  |  |  |  |  |  |
| S31 | Hα | 4.75 |  |  |  |  | 0.21* |  |  |  |
|  | Hβ_2_ | 4.53 | **^_^** | **^_^** | **^_^** | **^_^** | 0.22 | **^_^** | **^_^** | Mobile |
|  | Hβ_3_ | 4.23 |  |  |  |  |  |  |  |  |
|  |  |  |  |  |  |  |  |  |  |  |
| A32 | Hα | 4.22 |  |  |  |  | 0.26 | 0.26 |  |  |
|  | Hβ_1_ | 1.53 | **^_^** | **^_^** | **^_^** | **^_^** | 0.27 | 0.31 | **^_^** |  |
|  |  |  |  |  |  |  |  |  |  |  |
| D33 | Hα | 4.69 |  |  |  | 0.14 | 0.34* | 0.46 |  |  |
|  | Hβ_2_ | 2.98 | **^_^** | 0.43 | 0.87 | **^_^** | 0.54 | 0.53 | -5.85 | Gauche- |
|  | Hβ_3_ | 2.67 |  |  |  |  |  |  |  |  |
|  |  |  |  |  |  |  |  |  |  |  |
| G34 | Hα_2_ | 4.23 | **^_^** | **^_^** | **^_^** | 0.67 | 0.52 | 0.59 | -5.34 |  |
|  | Hα_3_ | 3.79 |  |  |  |  |  |  |  |  |
|  |  |  |  |  |  |  |  |  |  |  |
| R35 | Hα | 4.34 |  |  |  |  |  |  |  |  |
|  | Hβ_2_ | 1.96 | **^_^** | **^_^** | **^_^** | 0.32 | **^_^** | 0.63 | **^_^** | Mobile |
|  | Hβ_3_ | 2.05 |  |  |  |  |  |  |  |  |
|  |  |  |  |  |  |  |  |  |  |  |
| V36 | Hα | 4.71 |  |  |  |  | 0.31 | 0.47 |  |  |
|  | Hβ | 2.01 | **^_^** | **^b^** | **^_^** | 0.23 | 0.24 | 0.38 | **^_^** |  |
|  | Hγ_11_ | 0.81 |  |  |  |  | 0.22 |  |  |  |
|  | Hγ_21_ | 1.07 |  |  |  |  | 0.23 |  |  |  |
|  |  |  |  |  |  |  |  |  |  |  |
| Y37 | Hα | 4.84 |  |  |  | 0.52 | **^_^** | 0.52 |  |  |
|  | Hβ_2_ | 2.46 | 0.39 | 0.89 | 0.95 | **^_^** | 1.00 | 1.00 | -8.76 | Gauche- |
|  | Hβ_3_ | 2.79 |  |  |  |  |  |  |  |  |
|  |  |  |  |  |  |  |  |  |  |  |
| Y38 | Hα | 5.27 |  |  |  | 0.37 | 0.53 | 0.54 |  |  |
|  | Hβ_2_ | 2.69 | 0.88 | 0.90 | **^_^** | 0.75 | 0.73 | 0.93 | -6.40 | Gauche+ |
|  | Hβ_3_ | 2.94 |  |  |  |  |  |  |  |  |
|  |  |  |  |  |  |  |  |  |  |  |
| F39 | Hα | 5.62 |  |  |  | 0.53 | 0.52 | 0.57 |  |  |
|  | Hβ_2_ | 2.89 | 0.60 | 0.31 | 0.94 | 0.84 | 0.93 | 0.78 | -7.98 | Trans |
|  | Hβ_3_ | 2.61 |  |  |  |  |  |  |  |  |
|  |  |  |  |  |  |  |  |  |  |  |
| N40 | Hα | 4.44 |  |  |  | 0.44 | 0.43 | 0.59 |  |  |
|  | Hβ_2_ | -0.60 | 1 | 0.31 | 1 | 1.00 | 0.65 | 0.84 | -6.97 | Trans |
|  | Hβ_3_ | 2.00 |  |  |  |  |  |  |  |  |
|  |  |  |  |  |  |  |  |  |  |  |
| H41 | Hα | 4.11 |  |  |  | **^_^** | **^_^** | **^_^** |  |  |
|  | Hβ_2_ | 3.30 | **^_^** | 0.81 | **^c^** | **^_^** | **^_^** | 0.62 | -2.15 | Gauche- |
|  | Hβ_3_ | 3.08 |  |  |  |  |  |  |  |  |
|  |  |  |  |  |  |  |  |  |  |  |
| I42 | Hα | 3.87 |  |  |  | **^_^** | 0.32 | 0.39 |  |  |
|  | Hβ | 2.02 | **^a^** | **^b^** | **^_^** | **^_^** | 0.46 | 0.42 | **^_^** |  |
|  | Hg_12_ | 1.32 |  |  |  |  |  |  |  |  |
|  | Hg_13_ | 1.02 |  |  |  |  | 0.51 |  |  |  |
|  | Hγ_21_ | 0.79 |  |  |  |  | 0.30 |  |  |  |
|  | Hδ_11_ | 0.76 |  |  |  |  | 0.15 |  |  |  |
|  |  |  |  |  |  |  |  |  |  |  |
| T43 | Hα | 4.12 |  |  |  | 0.23 | 0.30* | 0.35 |  |  |
|  | Hβ | 4.25 | **^a^** | 0.84 | **^b^** | **^_^** | 0.40 | 0.28 | **^_^** |  |
|  | Hγ_21_ | 0.96 |  |  |  |  | 0.26 |  |  |  |
|  |  |  |  |  |  |  |  |  |  |  |
| N44 | Hα | 4.12 |  |  |  | 0.15 | 0.22 | 0.35 |  |  |
|  | Hβ_2_ | 3.14 | 0.51 | 0.69 | 0.44 | 0.48 | 0.28 | 0.37 | 0.54 | Average between Trans and Gauche+ |
|  | Hβ_3_ | 2.92 |  |  |  |  |  |  |  |  |
|  |  |  |  |  |  |  |  |  |  |  |
| A45 | Hα | 4.48 |  |  |  | 0.10 | 0.17 | 0.35 |  |  |
|  | Hβ_1_ | 1.26 | **^a^** | **^_^** | **^_^** | 0.22 | 0.26 | 0.26 | **^_^** |  |
|  |  |  |  |  |  |  |  |  |  |  |
| S46 | Hα | 6.01 |  |  |  | 0.40 | 0.43 | 0.50 |  |  |
|  | Hβ_2_ | 3.84 | **^_^** | 0.46 | 0.40 | 0.43 | 0.67 | 0.47 | 0.54 | Mobile |
|  | Hβ_3_ | 3.79 |  |  |  |  |  |  |  |  |
|  |  |  |  |  |  |  |  |  |  |  |
| Q47 | Hα | 4.60 |  |  |  | 0.19 | 0.48 | 0.44 |  |  |
|  | Hβ_2_ | 2.24 | **^_^** | 0.62 | 0.79 | **^_^** | **^_^** | 0.68 | -12.02 | Gauche- |
|  | Hβ_3_ | 2.56 |  |  |  |  |  |  |  |  |
|  | Hγ_2_ | 2.05 |  |  |  |  | 0.59 |  |  |  |
|  | Hγ_3_ | 2.00 |  |  |  |  |  |  |  |  |
|  |  |  |  |  |  |  |  |  |  |  |
| W48 | Hα | 4.96 |  |  |  | 0.14 | 0.44 | 0.47 |  |  |
|  | Hβ_2_ | 3.22 | 0.82 | 0.92 | 0.30 | 0.70 | 0.62 | 0.70 | -9.03 | Gauche+ |
|  | Hβ_3_ | 3.67 |  |  |  |  |  |  |  |  |
|  |  |  |  |  |  |  |  |  |  |  |
| E49 | Hα | 4.48 |  |  |  | 0.27 | 0.33 | **^_^** |  |  |
|  | Hβ_2_ | 1.91 | **^_^** | 0.68 | **^_^** | 0.49 | 0.36 | **^_^** | **^_^** | Mobile |
|  | Hβ_3_ | 1.91 |  |  |  |  |  |  |  |  |
|  | Hγ_2_ | 2.34 |  |  |  |  | 0.13 |  |  |  |
|  | Hγ_3_ | 2.27 |  |  |  |  |  |  |  |  |
|  |  |  |  |  |  |  |  |  |  |  |
| R50 | Hα | 4.69 |  |  |  | 0.21 | **^_^** | **^_^** |  |  |
|  | Hβ_2_ | 1.45 | **^_^** | **^_^** | **^_^** | **^_^** | 0.60 | **^_^** | **^_^** |  |
|  | Hβ_3_ | 1.45 |  |  |  |  |  |  |  |  |
|  | Hγ_2_ | 1.03 |  |  |  |  | 0.55 |  |  |  |
|  | Hγ_3_ | 1.24 |  |  |  |  |  |  |  |  |
|  | Hδ_2_ | 3.04 |  |  |  |  | 0.37 |  |  |  |
|  | Hδ_3_ | 3.04 |  |  |  |  |  |  |  |  |
|  |  |  |  |  |  |  |  |  |  |  |
| P51 | Hα | 3.93 |  |  |  |  | -0.02* | 0.38 |  |  |
|  | Hβ_2_ | 0.84 | 0.61 | **^_^** | **^_^** | **^_^** | 0.47 | 0.49 | -2.87 | Mobile |
|  | Hβ_3_ | 0.63 |  |  |  |  |  |  |  |  |
|  | Hγ_2_ | -0.04 |  |  |  |  | 0.50 |  |  |  |
|  | Hγ_3_ | 0.59 |  |  |  |  |  |  |  |  |
|  | Hδ_2_ | 2.44 |  |  |  |  | 0.80 |  |  |  |
|  | Hδ_3_ | 2.31 |  |  |  |  |  |  |  |  |
|  |  |  |  |  |  |  |  |  |  |  |
|  |  |  |  |  |  |  |  |  |  |  |
| S52 | Hα | 4.33 |  |  |  | 0.25 | 0.19 | 0.29 |  |  |
|  | Hβ_2_ | 3.81 | 0.36 | 0.30 | 0.45 | 0.49 | 0.35 | 0.26 | -1.70 | Mobile |
|  | Hβ_3_ | 3.75 |  |  |  |  |  |  |  |  |
|  |  |  |  |  |  |  |  |  |  |  |
| G53 | Hα_2_ | 3.78 | **^_^** | **^_^** | **^_^** | 0.11 | 0.15 |  | **^_^** |  |
|  | Hα_3_ | 3.78 |  |  |  |  |  |  |  |  |

^a^ Single Hβ peak, but no second Hβ peak for quantitative ^3^J coupling estimation.

^b^ Peak present in HNHB spectrum, but overlapped signal in 2D reference spectrum prevents quantitative ^3^J coupling measurement.

^c^ Peak present in HNCOHB spectrum, but overlapped signal in 2D reference spectrum prevents quantitative ^3^J coupling measurement.

* Diagonal peak used to calculate relaxation rate

**Fig. S2** Normalized β-methylene ^1^H R_DIPSI2_ relaxation rates derived from 3D ^1^H-TOCSY-^15^N-HSQC (a) 3D ^1^H-TOCSY-^13^C-HSQC (b) CBCACONH (c) plotted against Pin1 sequence. In both figures, a value of one corresponds to a rigid side chain excluding methyl-containing residues. Error bars are displayed in the vertical axis. The plot of ^1^H relaxation rates derived from 3D ^1^H-TOCSY-^15^N-HSQC versus ^1^H-TOCSY-^13^C-HSQC (d). Error bars are indicated in the vertical and horizontal axis

**Fig. S3** Normalized cross-correlated relaxation rates plotted as a function of Pin1 residues. A value of zero indicates a less restricted motion of a flexible methylene group

**(a)**

**(b)**

**Fig. S4** Normalized ^3^J-coupling experiments: ^3^J HαHβ (a), ^3^J HNCOHβ (b), and ^3^J HNHβ (c) plotted against the Pin1 sequence. Errors are indicated as vertical bars. These experiments provide a specific stereospecific assignment for the Cβ protons and define χ_1_ dihedral angle for a given residue if the side chain is restricted

**Fig. S5** The normalized maximum measured ^3^J-couplings (average of the two highest normalized values, which should in theory be the same) plotted against human Pin1 sequence. Errors are indicated with vertical bars—only data from amino acid residues with non-degenerate methylene Hβ chemical shifts were compared in this plot. A value of 1 corresponds to the most rigid residue in the protein


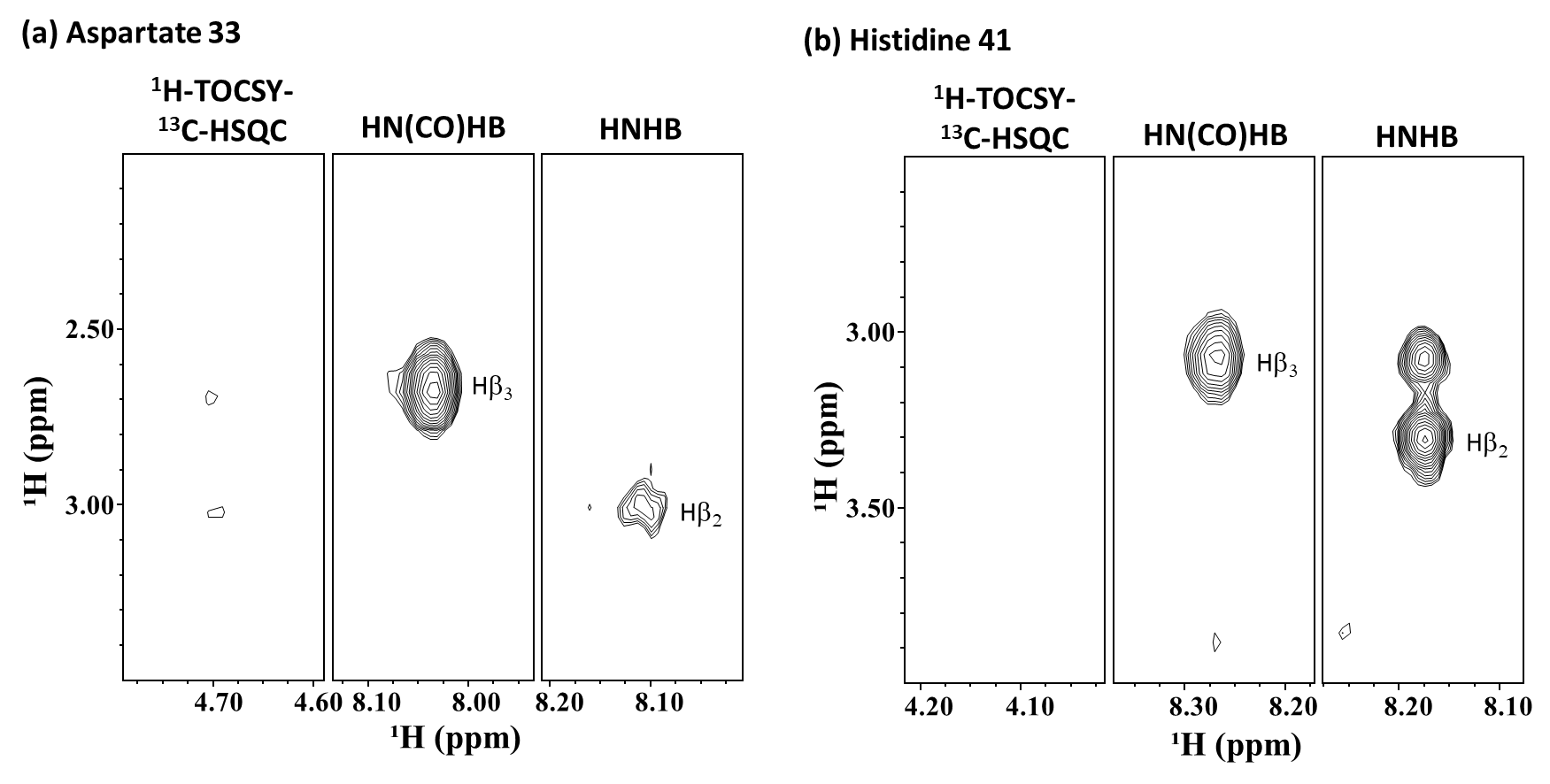


**Fig. S6** NMR strip plots from 3D ^1^H-TOCSY-^13^C-HSQC, HNCOHB, and HNHB experiments for Asp 33 (a) and His41 (b). Both residues adopt the gauche- χ_1_ rotamer with the upfield resonances assigned to the Hβ_3_. The strips of ^1^H-TOCSY-^13^C-HSQC and HNHB are taken at the Ha and NH frequency of residue-i, while HNCOHB strips are taken at the NH frequency of residue i+1
